# Tuning of NK-Specific HLA-C Expression by Alternative mRNA Splicing

**DOI:** 10.3389/fimmu.2019.03034

**Published:** 2020-01-10

**Authors:** Frederick J. Goodson-Gregg, Brian Rothbard, Amy Zhang, Paul W. Wright, Hongchuan Li, Victoria E. Walker-Sperling, Mary Carrington, Stephen K. Anderson

**Affiliations:** ^1^Cancer and Inflammation Program, Center for Cancer Research, National Cancer Institute, Frederick, MD, United States; ^2^Basic Science Program, Frederick National Laboratory for Cancer Research, Frederick, MD, United States; ^3^Ragon Institute of MGH, MIT, and Harvard, Cambridge, MA, United States

**Keywords:** human, HLA-C, NK, alternative splicing, NK-promoter, peptide loading

## Abstract

A complex system regulating HLA-C expression in NK cells, driven by an NK-specific promoter that produces alternatively spliced variants of the 5′-UTR has been recently identified. Exon content of the NK-specific 5′-UTR varies strikingly across *HLA-C* alleles, with some exons being allele specific. In order to investigate the possibility that allelic variation in the 5′-UTR modulates HLA-C expression levels, cDNAs containing several distinct classes of 5′-UTR were compared. Subtle changes in 5′-UTR content had a significant effect on the expression of HLA-C^*^03 and HLA-C^*^12 cDNA clones, suggesting that alternative splicing can fine-tune the level of protein expression. The HLA-C^*^06 allele was found to be highly expressed in relation to the other alleles studied. However, its increased expression was primarily associated with differences in the peptide-binding groove. Although the impact of allele-specific alternative splicing of NK-Pro transcripts on protein levels can be modest when compared with the effect of changes in peptide-loading, alternative splicing may represent an additional regulatory mechanism to fine-tune HLA-C levels within NK cells in distinct tissue environments or at different stages of maturation in order to achieve optimal levels of missing-self recognition.

## Introduction

Natural Killer (NK) cells are innate immune cells that sense transformed and virally infected cells using an array of activating receptors (1). The activation of NK cells is held in check by inhibitory receptors that recognize self MHC (2, 3). As NK cells mature, they shift from utilizing CD94:NKG2A receptors that recognize the invariant HLA-E molecule, to KIR that recognize specific subsets of HLA class I molecules (4, 5). All HLA-C alleles are recognized by at least one KIR, whereas less than half of the HLA-A or B alleles are KIR ligands (6). Notably, HLA-A and HLA-B surface protein levels are 13–18 times higher than HLA-C, further suggesting the main function of HLA-C is not the presentation of antigens, but rather it may be primarily a regulator of NK cell function (7). There is also variation in the expression level of individual HLA-C alleles, and increased levels of expression have been associated with improved outcomes in HIV infection (8). Several distinct mechanisms have been shown to affect HLA-C expression levels: polymorphisms in transcription factor binding sites; peptide loading efficiency; miRNA interaction with the 3′-UTR (9–11).

An NK-specific promoter (NK-Pro) has been identified in the *HLA-C* gene (12). The investigation of allelic variation in an Ets binding site 1.3 kb upstream of the HLA-C start codon led to the identification of a novel promoter that was shown to be NK cell specific. NK-Pro activity is associated with higher levels of HLA-C expression on mature NK cells. The *HLA-C* NK-Pro transcripts have highly variable 5′-UTR exon content generated by alternative splicing. The 5′-UTR consists of three non-coding exons, -1a, -1b, and -1c, as well as varying lengths of UTR upstream of the HLA-C start codon in exon 1 that result from differential splice acceptor sites (12). The NK-Pro may have evolved in order to modulate HLA-C levels in NK cells and regulate their lytic activity. The regulatory role of NK-Pro transcripts is supported by the observation of increased lytic activity of mature NK cells from individuals that are homozygous for *HLA-C* alleles that lack NK-Pro transcripts (4). In addition, the mRNA isoforms produced by the NK-Pro vary between immature and mature NK cells (12). Immature NK cells produce higher levels of splice variants that lack exon 1, and subsequently are not translatable, whereas mature NK cells produce lower levels of these exon skipping variants and have higher surface protein levels of HLA-C (12). This acquisition of higher levels of HLA-C driven by translatable NK-Pro transcripts corresponds with the acquisition of lytic activity, suggesting a regulatory role. Furthermore, the splice variants generated that do possess exon 1 have variable 5′-UTR lengths, resulting in variable translation efficiency, suggesting tuning of lytic activity by the NK-Pro via variation in HLA-C levels (12).

It has been previously shown that NK cell-intrinsic expression of HLA plays a role in NK cell education, and the level of HLA-C expression by NK cells is inversely correlated with their lytic activity (12, 13). Despite mounting evidence of *cis* interaction between KIR and HLA class I, direct binding within human NK cells has not yet been shown. Murine Ly49 have been shown to interact with class I MHC in *cis* due to a flexible stalk on the Ly49 protein (14). Furthermore, this *cis* interaction is required for murine NK cell licensing (15). KIR lack a flexible stalk, however KIR:HLA-C interaction could be occurring in endosomes. This *cis* interaction would account for the observed effect of HLA-C levels on NK cell lytic activity.

The allele-specific differences in 5′-UTR length and exon content implies that the NK-Pro evolved in order to modulate HLA-C expression in NK cells to produce optimal levels of inhibitory signaling. To investigate this possibility, the current study analyzed allele-specific differences in the NK cell expression level of HLA-C in individuals homozygous for *HLA-C* alleles with distinct patterns of exon usage, coupled with an analysis of the translatability of differentially-spliced *HLA-C* mRNAs. The results demonstrate that exon -1a/-1b/-1c content has an effect on the level of HLA-C protein expression, revealing an additional mechanism that may fine-tune HLA-C expression in developing NK cells in different tissue environments. The results also confirm a strong effect of variation in the peptide-binding groove of HLA-C alleles on their level of expression, as has been previously reported for the HLA-C^*^05 and HLA-C^*^07 alleles (11).

## Results

### *HLA-C* Homozygous Individuals Possess Distinct 5′-UTR Splicing Patterns

**Figure 1:**
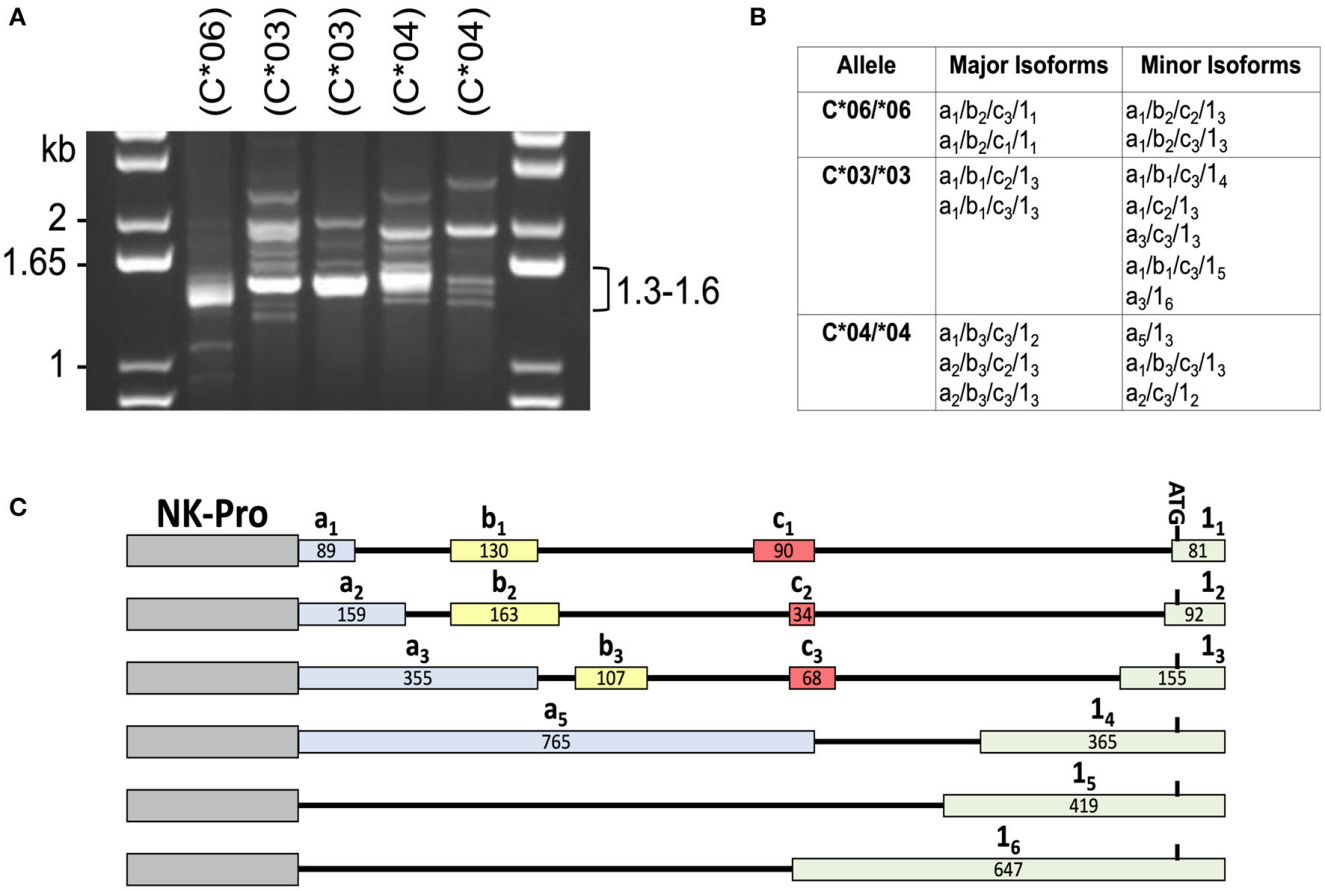
Individuals with homozygous *HLA-C* alleles exhibit distinct patterns of alternative transcripts. **(A)** PCR amplification of cDNA from individuals homozygous for specific *HLA-C* alleles. NK cell cDNA from *HLA-C* homozygous donors (C*06/*06; C*03/*03; C*04/*04) was amplified with exon -1a forward and exon 8 reverse primers. The size of bands in the DNA marker lanes (kb) and the size range of major bands amplified from the cDNAs is indicated. *HLA-C* allele is indicated as (C*##) above each lane. **(B)** Sequencing analysis of PCR products. A summary of the exon structure of translatable mRNA isoforms detected is shown. All isoforms listed contain exons 1–8 comprising the complete open reading frame. The alternative 5′-UTR exons a, b, c, and 1 are numbered as in Li et al. (12). **(C)** Summary of the *HLA-C* alternative exons observed in this study. The relative genomic position, name, and size in bp is shown for each of the exons present in the NK-Pro transcripts listed in **(B)**. The locations of the NK-Pro and the ATG initiation codon of the *HLA-C* gene are indicated.

In order to identify the patterns of 5′-UTR splicing for individual *HLA-C* alleles, we performed full-length RT-PCR on RNA isolated from purified peripheral blood NK cells from individuals that were homozygous for HLA-C^*^03, HLA-C^*^04, or HLA-C^*^06. We predicted that individuals of the same genotype would have similar splicing patterns, and this was the case for the major products observed in the 1.3–1.6 kb range (Figure 1A). Distinct patterns of minor bands were observed, which may represent variation in the tissue origin or maturation status of the peripheral blood NK cells between individuals. To identify the structures of these splice variants, the PCR products were cloned and sequenced. As previously described, allele-specific differences were present for the -1b exon and exon 1 (12). The number of clones sequenced (>100), was sufficient to identify the major and minor isoforms produced by each allele (Figure 1B). As predicted, the sizes of the major isoforms identified correspond to the strongest bands observed in the 1.3–1.6 kb region of the gel for each genotype. A summary of the exon variants detected is shown in Figure 1C. The allele-specific splice forms observed suggests the existence of an allele-specific regulatory mechanism for NK cell HLA-C expression.

### Alternative mRNA Splicing Results in Fine-Tuning of HLA-C Expression for HLA-C^*^03 and HLA-C^*^04

**Figure 2:**
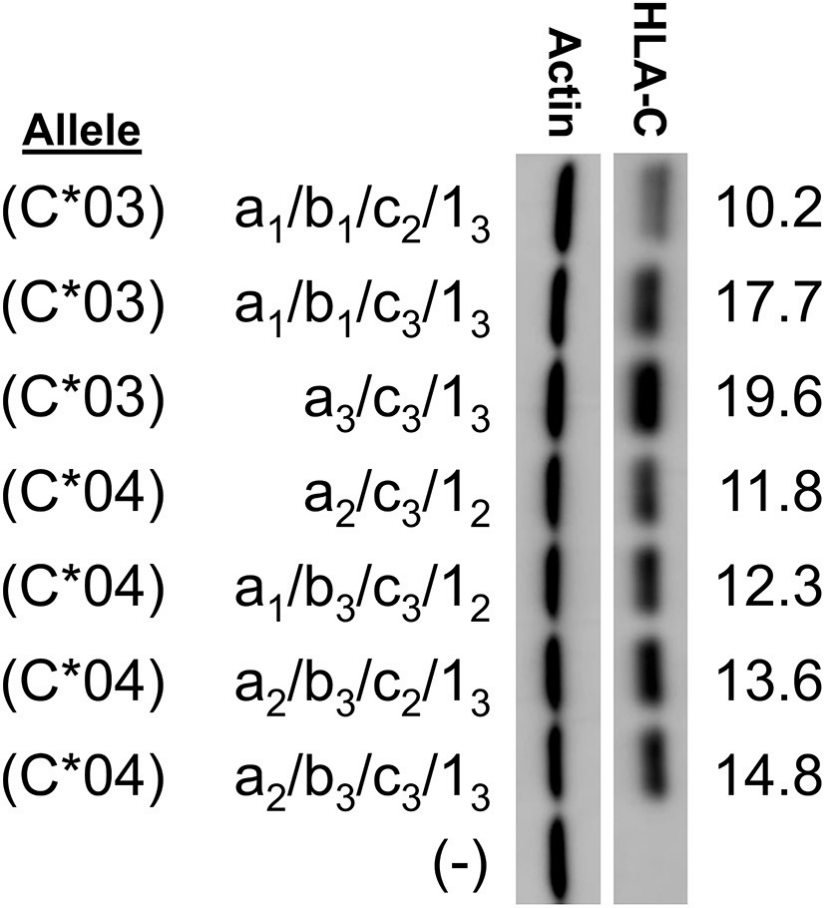
Western blotting of constructs for HLA-C*03 and HLA-C*04 indicate variation in protein expression due to alternative splicing. *HLA-C* allele is indicated at left. The exons present in each splice variant are indicated as per the format a/b/c/1-. HLA-C band intensity was quantified and normalized against actin intensity utilizing ImageJ, shown at the right. The results shown are representative of three independent experiments performed.

In order to examine the effect of the 5′-UTR on HLA-C expression levels, the human JAR trophoblast cell line, which does not express HLA-C, was transfected with full-length cDNA expression constructs containing distinct combinations of 5′-UTR exons. In order to control for any possible impact of the coding region on protein expression levels, panels of clones were selected that allowed for comparison within alleles. This strict analysis of the impact of the 5′-UTR compared similar length constructs for HLA-C^*^03 and HLA-C^*^04. Interestingly, despite identical coding regions and similar overall 5′-UTR length, HLA-C levels vary strikingly within alleles. For HLA-C^*^03 there was a nearly two-fold difference in expression between similar length constructs (Figure 2).

Remarkably, subtle changes in exon size in HLA-C^*^03 splice variants resulted in substantial changes in expression. The 5′-UTR sequences were compared with regard to the presence of potential competing initiation codons or RNA secondary structure that might affect their translatability (16). Several elements were considered: 5′-UTR length, competing ORF length, competing ORF proximity to the HLA-C coding region ORF, mRNA secondary structure, ribosome initiation interference as estimated by GC content, and variations in Kozak sequence (16). Splice forms in our initial panel contained variation in all of the aforementioned elements. While this initial panel indicated fine-tuning effects of the splice forms on HLA-C expression, this panel had too many competing mRNA elements to identify causative elements.

**Figure 3:**
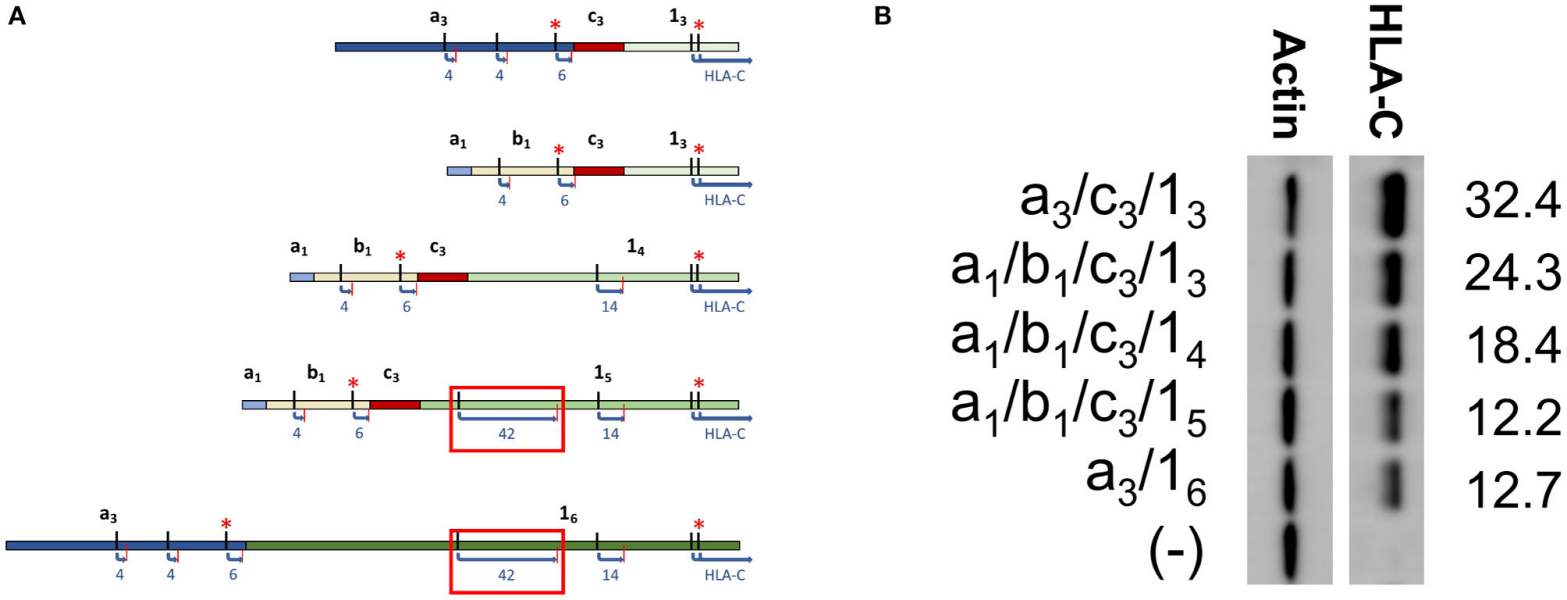
Western blotting of HLA-C*03 constructs containing exon 1 splice forms of increasing length indicate inclusion of a long ORF reduces protein expression. **(A)** Schematic illustrating splice forms utilized for the Western blot panel. Exons are indicated in bold. ORF start and stop codons are indicated by black and red lines, respectively. ORF Length is shown as blue text. Kozak sequence strength is indicated by the red asterisks, where at least one Kozak sequence enhancing nucleotide is present. The red box indicates the ORF associated with lower expression of a_1_/b_1_/c_3_/1_5_ and a_3_/1_6_. **(B)** Western blot comparing HLA-C*03 constructs with increasing exon 1 length. Alternative exons present in each construct are indicated on the left. ImageJ normalized lane intensities are indicated on the right. The results shown are representative of three independent experiments performed.

In order to directly test if the inclusion of a long competing ORF reduced protein expression, a panel of HLA-C^*^03 cDNAs with varying exon 1 length was examined. Exon 1 splice forms contain the same 3′ splice donor site, with alternative splice acceptor sites increasing the 5′-UTR length (Figure 3A). Analysis of sequence data for HLA-C^*^03 indicated that exon 1_5_ vs. 1_4_ contained a competing 42 amino acid ORF close to the start codon of the HLA-C protein (Figure 3A). As per our expectations, there was a decrease in protein expression with the inclusion of the 42 amino acid competing ORF, as seen by comparing lanes 1–3 with 4–5 (Figure 3B).

### The HLA-C^*^06 Allele Is Highly Expressed When Compared With the Expression of HLA-C^*^03 and HLA-C^*^04

**Figure 4:**
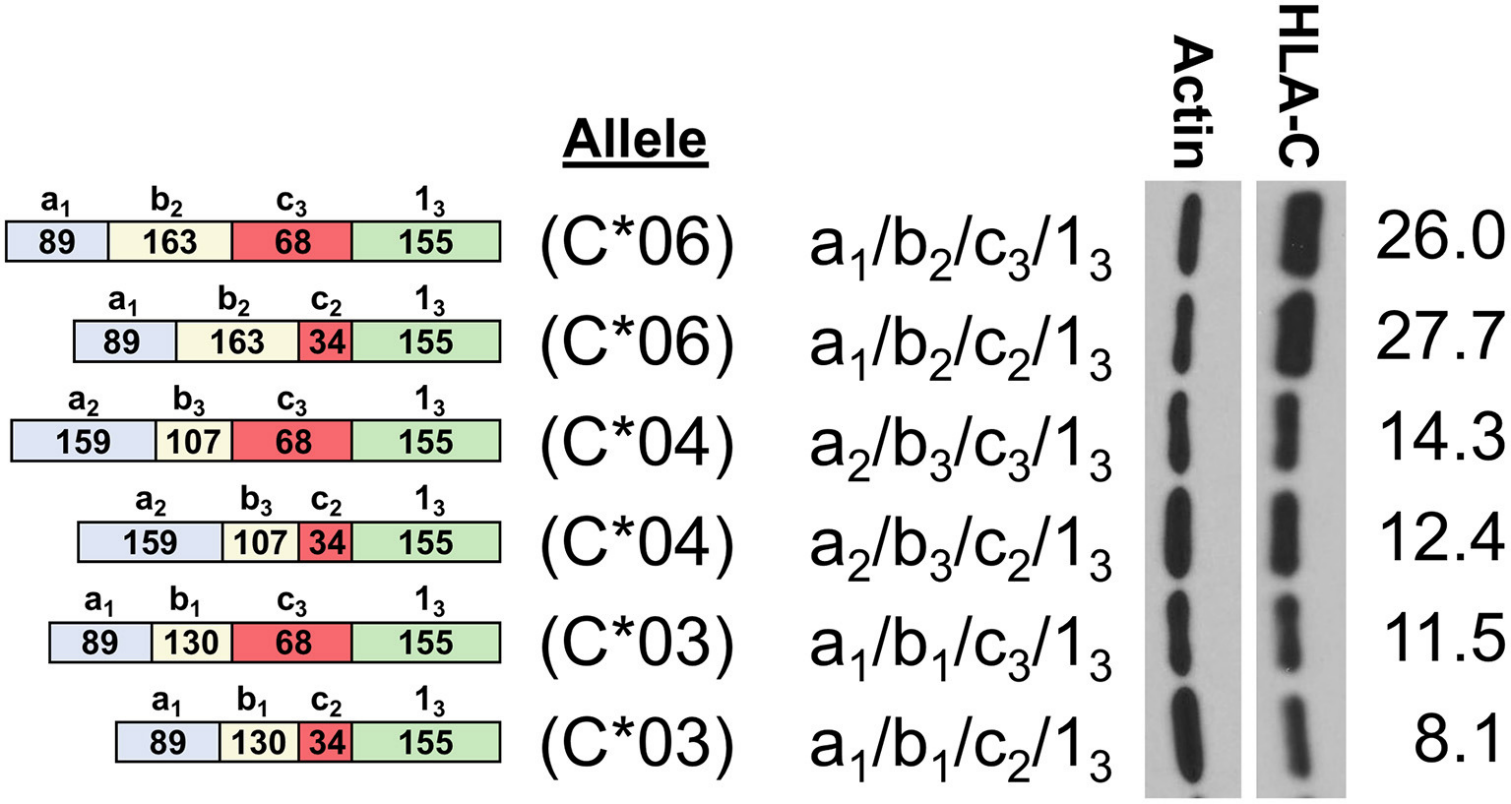
HLA-C*06 is expressed at markedly higher levels than -C*03 and -C*04 alleles. The exon content of each HLA-C cDNA is listed and shown schematically on the left. The results of Western blotting of transfected JAR cells is shown in the panel on the right. ImageJ normalized lane intensities are indicated on the right. The results shown are representative of three independent experiments performed.

In order to assess the impact of the coding region of *HLA-C* as well as allele-specific exons on HLA-C expression, cDNAs for HLA-C^*^03, HLA-C^*^04, and HLA-C^*^06 were compared by Western blot. Interestingly, the -1b_2_ exon specific to HLA-C^*^06 and -C^*^12 initially appeared to be associated with markedly higher levels of protein expression relative to the HLA-C^*^03 allele containing exon -1b_1_ when isoforms that are identical for the other 5′-UTR exons were compared (Figure 4). However, because the 5′-UTR and coding region vary simultaneously when comparing HLA-C^*^06 to HLA-C^*^03 and HLA-C^*^04, an additional panel was necessary to assess their relative influences on protein expression.

### Expression Patterns of HLA-C^*^12 mRNA Isoforms Show an Effect of Exon -1c and Exon 1 Size on Expression Levels Together With Coding Region Effects

**Figure 5:**
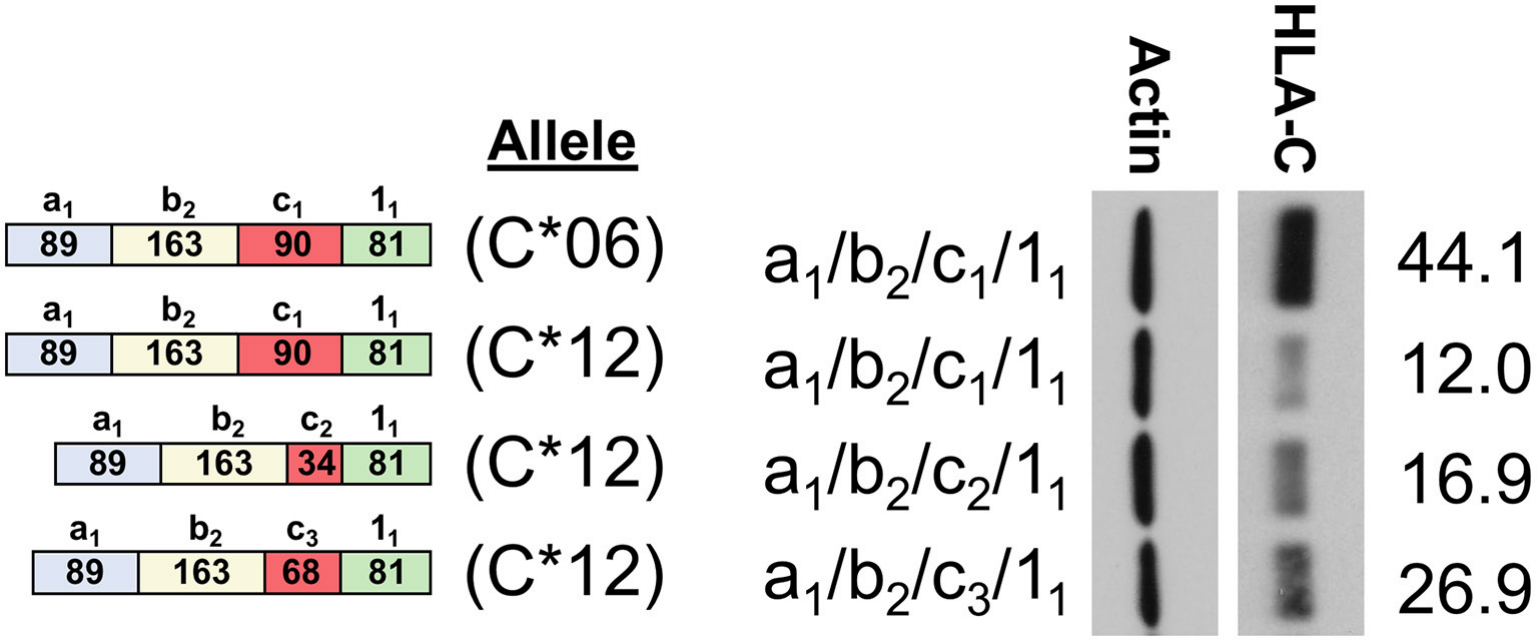
Western Blot comparing HLA-C*06 and HLA-C*12. The 5′-UTR structure of clones and a schematic representation of exon content for each structure is shown on the left. HLA-C allele is indicated as C*06 or C*12. The 5′-UTR is also indicated on the left, immediately adjacent to the Western blot. ImageJ actin normalized HLA-C intensity is indicated rightmost. The results shown are representative of three independent experiments performed.

To assess the relative impact of the coding region vs. the 5′-UTR of *HLA-C*, an additional panel of cDNA expression clones was generated for the HLA-C^*^12 allele. This allele contains a 5′-UTR that is identical to HLA-C^*^06, thereby allowing a direct assessment of coding region effects. The 5′-UTRs of the HLA-C^*^06 and HLA-C^*^12 cDNAs examined in lanes 1 and 2 of Figure 5 are identical, indicating that the coding region of HLA-C^*^06 drives markedly higher expression. This is of particular interest, since the coding regions of HLA-C^*^12 and HLA-C^*^06 vary by only 5 amino acids in exon 2, and HLA-C^*^06 exhibits markedly higher expression. Additionally, the coding region of all HLA-C^*^12 constructs shown are identical, allowing a direct assessment of the effect of the 5′-UTR on expression. When holding constant the coding region of HLA-C^*^12, differences in the 5′-UTR content appear to significantly impact expression, demonstrating that the 5′-UTR indeed has an important regulatory impact as shown by lanes 2–4. Thus, it appears that for HLA-C^*^03 and HLA-C^*^12, while the variable exons present in the 5′-UTR of *HLA-C* do impact protein expression levels significantly, this effect is hidden when comparing structures across alleles due to the larger impact of the coding region.

### Assessment of the Effect of Peptide Loading on Expression

We sought to determine why HLA-C^*^06 has markedly higher levels of protein expression when compared with C^*^03, C^*^04, and C^*^12. As previously identified when comparing HLA-C^*^06 and HLA-C^*^12, it appeared that amino acid changes present in exon 2 drive the difference in expression. Interestingly, it has been previously shown that exons 2/3, which are associated with peptide loading, can drive a higher level of expression for HLA-C^*^05 when compared with HLA-C^*^07 (11). To assess if differences in the peptide-binding pocket are responsible for the difference between HLA-C^*^03 and HLA-C^*^06 expression, we carried out a temperature-dependent expression analysis comparing HLA-C^*^03 and HLA-C^*^06 at 28°C and 37°C.

**Figure 6:**
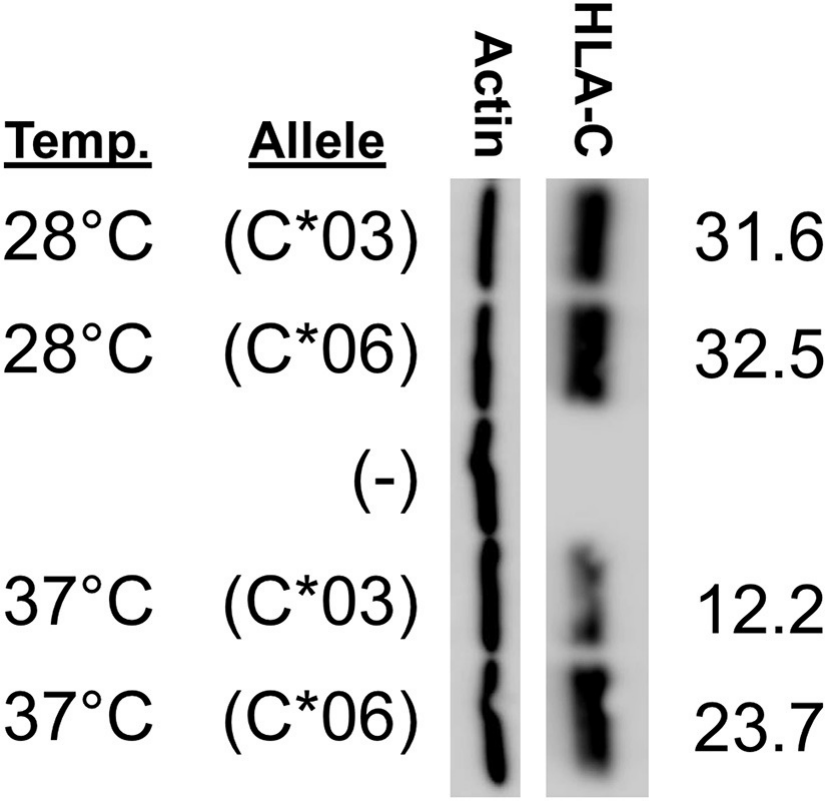
HLA-C*03 and -C*06 expression is similar at low temperature. Growth temperature of JAR cells transfected with HLA-C expression constructs is indicated on the left. The transfected HLA-C*03 a_1_/b_1_/c_2_/1-3 cDNA is indicated as (C*03) and the a_1_/b_2_/c_2_/1-3 HLA-C*06 cDNA is indicated as (C*06), (–) represents cells transfected with a negative control vector. ImageJ actin-normalized HLA-C intensities are quantified and shown at right. Results are reflective of three independent experiments.

We anticipated that lower temperature would improve the affinity of the HLA-C^*^03 binding pocket for peptides, and that HLA-C^*^06 would no longer be significantly higher at this lower temperature. Consistent with expectations, at 28°C, the difference between HLA-C^*^03 and HLA-C^*^06 expression was abolished, suggesting that at lower temperatures both are loaded with peptide and transported to the cell surface at similar rates and this affects protein stability and subsequent expression levels (Figure 6).

### *HLA-C* Alleles Exhibit Significantly Different NK Cell Expression Levels

**Figure 7:**
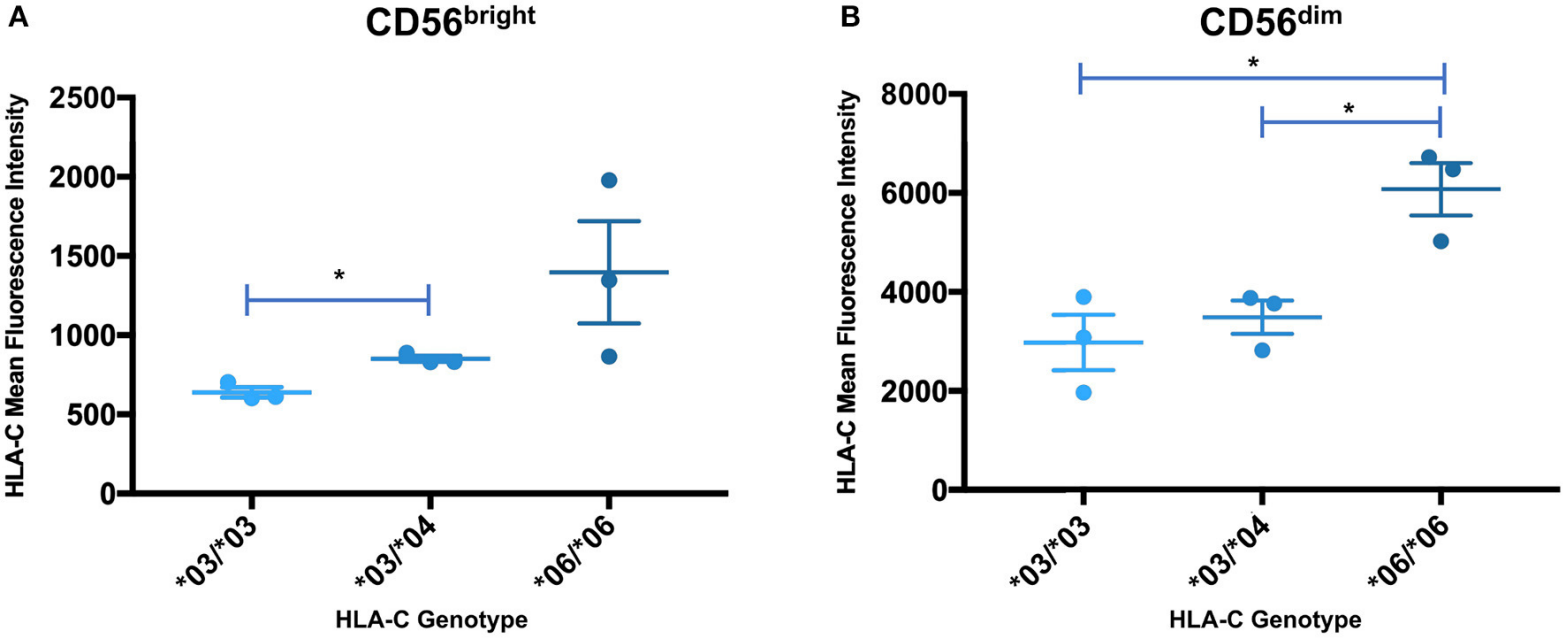
Expression levels of HLA-C alleles on peripheral blood NK cells are consistent with the expression level observed by Western blot analysis. The mean fluorescence intensity of HLA-C expression on peripheral blood NK cells is shown for donors that are homozygous for HLA-C*03 (*03/*03) or HLA-C*06 (*06/*06), and heterozygous individuals possessing HLA-C*03 and HLA-C*04 (*03/*04). Statistical significance (*p*-value < 0.05) is indicated by the asterisks (unpaired *t*-test). **(A)** HLA-C intensity for CD56^bright^ NK cells. **(B)** HLA-C intensity for CD56^dim^ NK cells. The *06/*06 individuals have a significantly higher level of HLA-C expression than either the *03/*03 or *03/*04 individuals (*03/*03 vs. *06/*06 *p* = 0.016, *03/*04 vs. *06/*06 *p* = 0.0146, unpaired *t*-test).

In order to determine if the trend identified by Western blotting for the relative levels of HLA-C^*^03/^*^04/^*^06 was present in circulating NK cells, peripheral blood mononuclear cells were isolated from whole blood acquired from healthy donors and analyzed by flow cytometry. Measurements of different panels of HLA-C^*^03/^*^04/^*^06 donors in separate experiments yielded the same trend observed in Western blotting experiments for both immature and mature NK cells. While the same trend observed by Western blotting is present for immature CD56^bright^ cells, the differences supporting the trend are not statistically significant (Figure 7A). However, mature CD56^dim^ NK cells exhibit the same trend with statistically significant differences, consistent with the previously observed upregulation of HLA-C on mature CD56^dim^ NK cells (Figure 7B) (8). There is an approximately two-fold increase in mean fluorescence intensity (MFI) between genotypes containing HLA-C^*^03 or HLA-C^*^04, and the HLA-C^*^06 homozygotes (Figure 7B). This provides novel evidence that in vivo, HLA-C^*^06 has significantly higher surface protein level expression on NK cells.

## Discussion

The previous identification of an *HLA-C* NK-specific promoter capable of generating a wide array of alternatively spliced mRNAs, as well as allele-specific splice variants, suggested that endogenous HLA-C expression may play an important role in NK cell activity or education (12). Our previous work identified preferential generation of untranslatable transcripts in immature NK cells, and the existence of HLA-C alleles in which the NK-Pro is not active due to a single nucleotide polymorphism that disrupts a key transcription factor-binding site (12). Here we perform a thorough characterization of the allele-specific differences in the 5′-UTR content of *HLA-C* mRNA transcripts originating from the NK-Pro. We speculated that variation in HLA-C expression levels across alleles would represent a mechanism to tune NK-cell lytic activity. We predicted that differences in the exon content of the 5′-UTR would drive marked differences in HLA-C expression on NK cells. Our analyses holding the coding region constant and comparing NK-Pro transcripts within alleles for HLA-C^*^03, HLA-C^*^04, and HLA-C^*^12 indicate that, indeed, 5′-UTR exon content has an impact on HLA-C levels (Figures 2, 3B, 5). These results reveal an additional mechanism that can control the level of HLA-C expression on NK cells. We had previously shown that the 5′-UTR length inversely correlates with protein expression level (12). However, here we have observed a multitude of mRNA sequence elements that can have significant impacts on protein expression.

It has previously been shown that ribosome initiation is inhibited by mRNA secondary structure in the 5′-UTR (17), and that GC-rich sequence proximal to the 5′ end can block ribosome initiation (18–20). Interestingly, the structures of C^*^03 a_3_/c_3_/1_3_ and C^*^03 a_1_/b_1_/c_3_/1_3_ vary only by the inclusion of a 136 bp intron spanning the genomic space between the splice donor site of exon -1a_1_ and the splice acceptor site of -1b_1_: however, the larger C^*^03 a_3_/c_3_/1_3_ splice form is expressed at a higher level (Figure 3A). The intronic region has a GC content of 38%, which is substantially lower than the surrounding region (48–58% GC). Furthermore, RNA-fold analysis of predicted structures indicated that the 5′ end of the mRNA containing the intronic region was not base-paired, as compared to mRNA structures without the sequence (21, 22). Additionally, this region offers an explanation for the unexpected similarity of expression between C^*^03 a_3_/1_6_ and C^*^03 a_1_/b_1_/c_3_/1_5_. Our previous investigation of the impact of 5′-UTR length suggested that additional sequence reduced expression, likely owing to additional interfering secondary structure and ribosomal scanning effects (12, 17). However, despite C^*^03 a_3_/1_6_ being larger than C^*^03 a_1_/b_1_/c_3_/1_5_ by 296 base pairs, they have similar expression levels. We speculate that the potential negative effects of an additional 160 base pairs included when comparing exon 1_6_ to 1_5_ might be offset by the secondary structure effects of the lower GC content of the 136 base pairs present in the a_3_ exon enhancing ribosomal initiation. Indeed, the numerous, and sometimes oppositional, mRNA elements present across these various splice forms offer a multitude of evolutionary targets for achieving distinct levels of HLA-C protein expression in NK cells.

The analysis presented here supports and extends previous findings on the role of the 5′-UTR in HLA-C expression. The full unspliced 1.3 kb UTR present in the -1a_7_ NK-Pro transcript contains 15 potential competing start codons in the region prior to the start of the HLA-C coding region in exon 1, which could account for the weak translation of this mRNA isoform relative to the shorter spliced isoforms (12). However, only three of the potential start codons contain additional flanking nucleotides that contribute to efficient translation initiation [**RCC**AUG**G**; (23)], and the first two AUGs are associated with short 3-5 amino acid open reading frames, which would allow continued scanning of the mRNA and reinitiation (24). The third CCAUGG element is of particular interest, as it is located immediately prior to the splice donor sequence used to generate the -1c_1_ and -1c_2_ exons. Splicing of exon -1c_1/_c_2_ to exon 1_1_ results in an open reading frame that spans the HLA-C AUG, potentially reducing HLA-C expression if this alternative AUG is used. The observed expression patterns of HLA-C^*^12 isoforms are consistent with this hypothesis: cDNAs containing exons -1c_1/_c_2_ spliced to exon 1_1_ are poorly expressed relative to the cDNA containing exon -1c_3_ (Figure 5). It is important to note that the longer -1c_3_ and -1c_4_ exons contain a T nucleotide following this sequence (CCAUGG**T**) that negates the initiation-enhancing effect of a G at position +4 (25). Therefore, it appears that the variation in the size of the -1c exons and the exon 1 splice acceptor used can directly affect the efficiency of HLA-C translation. Although the relatively large impact of the coding region on HLA-C levels precludes meaningful comparison between alleles, the examination of the effect of 5′-UTR sequence variation on the expression of individual alleles (HLA-C^*^03, HLA-C^*^04, and HLA-C^*^12) strongly supports the role of the NK-Pro 5′-UTR in modulating HLA-C levels on NK cells and provides further evidence that precise control of endogenous HLA-C expression is important for NK cell development and function.

The impact of the coding region on HLA-C expression level was striking. A comparison of HLA-C^*^12 to HLA-C^*^06, which have identical 5′-UTRs, reveals that the coding region can account for large differences in expression (Figure 5). The high level of HLA-C^*^06 expression on NK cells has not been previously described, and the flow cytometry analysis of *ex vivo* NK cells verified the relevance of expression levels measured by Western blotting of transfected cells (Figure 7). Furthermore, a re-examination of the effect of HLA-C levels on NK cell activity revealed that individuals possessing the HLA-C^*^06 allele consistently had the lowest activity (12). This observation predicts that the NK cell-mediated anti-leukemia effect would be greatest in individuals with *HLA-C* alleles lacking NK-Pro activity (HLA-C^*^02, -C^*^05, -C^*^07, -C^*^08, -C^*^17) and lowest in individuals that possess HLA-C^*^06 together with the C2-binding KIR2DL1 inhibitory receptor, a factor that may be significant when choosing donors for stem cell transplantation. Although the presence of specific HLA-C alleles and their cognate receptors is important for the process of education or licensing that affects the development of fully functional NK cells, the control of HLA-C expression levels by variation in the structure and translatability of NK-Pro transcripts likely reflects a tuning mechanism that modulates the sensitivity of missing-self recognition. With regard to identifying the amino acid residues that are responsible for the high expression of HLA-C^*^06, the high degree of homology between HLA-C^*^06 and HLA-C^*^12 allows for a more precise localization of the changes responsible, as there are only 5 amino acid differences between the two alleles, and all are in exon 2. Exon 2 and exon 3 of *HLA-C* encode the peptide-binding groove of the protein, and previous work by Kaur et al. (11) demonstrated that the difference in expression between the HLA-C^*^05 and HLA-C^*^07 alleles was due to differences in exons 2 and 3. Peptide loading and transport to the cell surface is believed to govern protein stability, as protein that is not efficiently translocated to the surface is degraded (26). If the higher expression of HLA-C^*^06 is due to more efficient peptide loading, then these differences should be minimized if cells are grown at a lower temperature, facilitating peptide loading of the less efficient alleles (27). Western blotting of transfected cells grown at 28°C resulted in nearly equivalent levels of HLA-C^*^03 and HLA-C^*^06 expression. This confirmed a key role for the peptide-binding domain for HLA-C expression, as previously demonstrated for HLA-C^*^05 and HLA-C^*^07 (Figure 6).

It is of interest to note that the highly expressed HLA-C^*^05 and the weakly expressed HLA-C^*^07 alleles previously analyzed both possess a polymorphism in the NK-Pro element that abrogates its activity, and these alleles are not highly expressed by NK cells. While polymorphisms in the NK-specific promoter and NK-specific 5′-UTR exons modulate expression of HLA-C in NK cells, polymorphism in the peptide-binding groove will affect surface expression of HLA-C in all cells. Therefore, there has been evolutionary selection for changes in the efficiency of HLA-C presentation of peptides to T cells concurrent with selection for changes in the NK cell-intrinsic expression of HLA-C. Although the primary evolutionary force driving the emergence of the *HLA-C* gene was likely the provision of ligands for KIR, the ability to present peptides is important for two reasons: first, KIR bind to the distal end of the peptide-binding groove, and the affinity for HLA is modulated by the peptide present (28); second, presentation of viral peptides by HLA-C is necessary to ensure that viruses maintain mechanisms that down-regulate HLA-C to avoid T cell recognition, making infected cells susceptible to missing-self recognition by NK cells. There are strain-specific differences in the ability of HIV to down-regulate HLA-C expression, suggesting that differences in the relative efficiency of HLA-C alleles to present peptide vs. inhibit NK cell activity may determine the survival benefit of HLA-C downregulation for the virus (29, 30).

In summary, the current study demonstrates that subtle changes in the 5′-UTR of NK-specific *HLA-C* transcripts can affect the level of protein expression. It will be of interest to determine if there are tissue-specific changes in 5′-UTR composition that regulate the level of NK cell HLA-C, and thus their relative lytic activity in different tissues. We also show that HLA-C^*^06 has markedly higher expression levels than HLA-C^*^03, HLA-C^*^04, and HLA-C^*^12, and high HLA-C^*^06 expression is associated with amino acid variation in the peptide-binding groove, consistent with previous results comparing HLA-C^*^05 and -C^*^07 expression. The consideration of the presence of *HLA-C* alleles with high vs. low expression on NK cells may be of clinical benefit when choosing donors for stem cell transplantation or adoptive transfer of NK cells.

## Materials and Methods

### JAR Cell Line

The JAR human trophoblast cell line was acquired from ATCC (Manassas, CA, USA), and was grown in RPMI 1640 media containing 10% fetal bovine serum, 100 U/ml penicillin, 100 U/ml streptomycin, and L-glutamine.

### Donors and NK Cell Isolation

Blood was obtained from healthy volunteers recruited through the NCI-Frederick Research Donor Program (http://ncifrederick.cancer.gov/programs/science/rdp/default.aspx). The *KIR* and *HLA* genotype for each donor was determined as previously described (21). NK cells were acquired from the peripheral blood of donors by Histopaque (Sigma-Aldrich, St Louis, MO, USA) gradient centrifugation utilizing the RosetteSep Human NK Enrichment Cocktail and SepMate tubes (STEM-CELL Technologies, Vancouver, BC, Canada).

### RT-PCR

Total RNA was isolated from NK cells purified from the peripheral blood of healthy volunteers, and cDNA synthesis was performed as previously described (8).

### Western Blotting of HLA-C Transfectants

Full length *HLA-C* cDNAs were PCR amplified utilizing GoTaq Long PCR Master Mix (Promega Corporation, Madison, WI) as previously described (4), gel extracted, TOPO cloned into the pEF6/V5-His TOPO-TA vector (Thermo Fisher Scientific, Waltham, MA, USA) and verified by sequencing. 5 ug of each construct were transfected into the human JAR trophoblast cell line utilizing HilyMax Transfection Reagent (Dojindo Molecular Technologies Inc., Rockville, MD, USA). Cells were harvested with Nonidet-P40 (NP-40) lysis buffer (1% NP-40, 50 mM Tris-HCL, pH 8.0, 150 mM NaCl) supplemented with complete mini protease inhibitor cocktail tablets (Roche Diagnostics, Indianapolis, IN, USA). Protein concentrations were determined using a Nanodrop 2000 spectrophotometer and BCA Protein Assay (Thermo Fisher Scientific). Thirty microgram of total protein were separated using sodium dodecyl sulfate-PAGE (SDS-PAGE) on 4–12% Tris-Glycine gels (Thermo Fisher Scientific) and transferred to Immobilon-P membrane (Sigma-Aldrich). Membranes were blocked 4°C overnight in 5% milk solution in PBST (Phosphate Buffered Saline, pH 7.4, 0.1% Tween 20) and were probed 1.5 h with anti-HLA-C antibody (Abcam, Cambridge, MA, USA; ab126722) diluted 1:1,000 in 5% milk PBST solution. Blots were washed 4 times for 5 min with PBST, then were probed for 20 min with anti-rabbit HRP-linked IgG (Cell Signaling Technology, Danvers, MA, USA) diluted 1:20,000 in 5% milk PBST solution. Blots were washed 4 times for 5 min with PBST, then visualized using Amersham ECL Western blotting detection reagents (GE Healthcare, Pittsburg, PA, USA). Protein loading was normalized via blots being stripped for 5 min using either Restore PLUS Western Blot Stripping Buffer (Thermo Fisher Scientific) or 7.5% hydrogen peroxide, then washed 3 times for 5 min with PBS. Blots were blocked at room temperature for 1 h in 5% milk PBST solution, then probed 1.5 h with monoclonal anti-β-actin antibody (Sigma-Aldrich A2228) diluted 1:2,000 in milk PBST solution. Blots were washed 4 times for 5 min in PBST, then probed with anti-mouse HRP linked IgG antibody (Cell Signaling Technology) diluted 1:20,000 in milk PBST solution for 20 min. β-actin levels were visualized with Amersham ECL Western blotting detection reagents (GE Healthcare). Lanes were quantified using ImageJ.

### Flow Cytometry of HLA-C Levels of Blood Donors

PBMCs were separated from whole blood using SepMate tubes (STEMCELL Technologies) with Histopaque (Sigma-Aldrich) gradient centrifugation. For each blood donor, 1,000,000 cells were stained at 4°C for 10 min with: Pacific Blue Mouse Anti-Human CD3 [clone UCHT1] (BD Biosciences, Franklin Lakes, NJ), Brilliant Violet 711™ anti-human CD56 (NCAM) Antibody [clone HCD56] (BioLegend, San Diego, CA), and PE Mouse Anti-Human HLA-C [clone DT9] (BD Biosciences). Sample data was collected utilizing a BD LSRFortessa flow cytometer and was analyzed with FlowJo v10.1. Unpaired *t*-tests of sample data were performed utilizing Prism 7.

## Data Availability Statement

The datasets generated for this study can be found in the GenBank MH254922-MH254937.

## Ethics Statement

Ethical review and approval was not required for the study on human participants in accordance with the local legislation and institutional requirements. The patients/participants provided their written informed consent to participate in this study.

## Author Contributions

FG-G and SA contributed to the conception and design of the study, analyzed the data, and wrote and edited the manuscript. FG-G, BR, AZ, PW, HL, and VW-S performed experiments and edited the manuscript. MC contributed to the editing of the manuscript and all authors approved the submitted version.

### Conflict of Interest

The authors declare that the research was conducted in the absence of any commercial or financial relationships that could be construed as a potential conflict of interest.
